# Associations of leptin and corticostriatal connectivity in bipolar disorder

**DOI:** 10.1038/s41598-022-26233-8

**Published:** 2022-12-19

**Authors:** Shyh-Yuh Wei, Huai-Hsuan Tseng, Hui Hua Chang, Wei Hung Chang, Yen Kuang Yang, Po See Chen

**Affiliations:** 1 Department of Psychiatry, National Cheng Kung University Hospital, College of Medicine, National Cheng Kung University, 138 Sheng Li Road, North Dist., 70403 Tainan, Taiwan; 2 Institute of Behavioral Medicine, College of Medicine, National Cheng Kung University, Tainan, Taiwan; 3 Institute of Clinical Pharmacy and Pharmaceutical Sciences, College of Medicine, National Cheng Kung University, Tainan, Taiwan; 4 School of Pharmacy, College of Medicine, National Cheng Kung University, Tainan, Taiwan; 5 Department of Pharmacy, National Cheng Kung University Hospital, College of Medicine, National Cheng Kung University, Tainan, Taiwan; 6 Department of Pharmacy, National Cheng Kung University Hospital, Dou-Liou Branch, Yunlin, Taiwan; 7 Department of Psychiatry, National Cheng Kung University Hospital, Dou-Liou Branch, Yunlin, Taiwan; 8 Department of Psychiatry, Tainan Hospital, Ministry of Health and Welfare, Tainan, Taiwan

**Keywords:** Bipolar disorder, Functional magnetic resonance imaging

## Abstract

Bipolar disorder (BD) and metabolic disturbance represent a chronic state of low-grade inflammation and corticostriatal circuitry alterations. Herein, we aimed to investigate whether plasma leptin, an adipokine that plays a key role in the interplay of metabolism and inflammation, is associated with corticostriatal connectivity in patients with BD. Twenty-eight BD I patients, 36 BD II patients and 66 healthy controls were enrolled and completed the Hamilton Depression Rating Scale, the Young Mania Rating Scale, and the Recent Life Change Questionnaire. Fasting plasma leptin and C-reactive protein (CRP) levels were measured, and corticostriatal connectivity was examined using functional magnetic resonance imaging (fMRI). The relationships between leptin, CRP and body mass index (BMI) identified in the controls and BD II patients were absent in the BD I patients. We did not find a significant group difference in the leptin level; nevertheless, the negative correlation between leptin level and corticostriatal connectivity (ventrolateral prefrontal cortex and inferior temporal gyrus) observed in the healthy controls was absent in the BD patients. The disproportionate increase in leptin level with increasing BMI in BD indicated a potential inflammatory role of white adipose tissue in BD. Furthermore, higher CRP levels in BD I patients might induce leptin resistance. Collectively, our results implied vulnerability to inflammatory and metabolic diseases in patients with BD, especially BD I.

## Introduction

Bipolar disorder (BD), characterized by emotion dysregulation, increases the relative risk of mortality^1^, which is partially mediated by metabolic disturbance^2–4^. Furthermore, evidence has shown that aberrant immune signaling contributes to all stages of BD, and also to metabolic disturbance^5,6^. Recent structural and functional studies have directly implicated reward system dysregulation in BD^7–9^, and evidence has shown that core network functional abnormalities lead to aberrant reward-processing, and the neural circuitry in BD is highly associated with systemic inflammation^10,11^. Such systemic inflammation might drive changes in dopaminergic corticostriatal circuitry connectivity^12,13^, and a recent large-scale study indicated that both BD and body mass index (BMI) were associated with similar regional brain volumes, including that of the basal ganglia^14^.

Leptin is secreted by white adipose tissue and regulates energy homeostasis. There is direct brain-adipose crosstalk through leptin, which regulates the energy balance and motivation through action at distinct neural circuits^15–18^. In addition, there is compelling evidence implicating leptin as an important modulator of the inflammatory process. Leptin can increase the production of pro-inflammatory cytokines and modulates both innate and adaptive immune responses^2,19,20^. Leptin itself has been found to be able to stimulate C-reactive protein (CRP) synthesis from the liver and endothelial cells^21^. A positive and independent relationship between peripheral leptin and CRP has been found previously in healthy subjects^22^. Taken together, leptin could modulate the reward system and plays a key role in the interplay of metabolism and inflammation^23^.

Both leptin and CRP are linked directly and independently through a number of pathophysiological mechanisms to BD^2,24–26^. However, whether the pathophysiological processes induced by leptin or CRP are enhanced or redundant in BD remains unknown^27^. We previously reported altered corticostriatal circuitry in BD that was associated with the level of CRP^28^. In the current study, we aimed to investigate the relationship between peripheral leptin and CRP levels. Regarding the role of the caudate nucleus in both metabolic control and BD^29^, we also investigated the possible correlation between leptin and caudate-seeded corticostriatal connectivity in patients with BD^30–32^. We hypothesized that the alterations in caudate-seeded functional connectivity (FC) in the corticostriatal circuitry may be linked to leptin metabolic feedback dysregulation in BD patients.

## Results

### Demographic and clinical data

Twenty-eight BD I patients, 36 BD II patients and 66 age-, sex-, life events-, and BMI-matched healthy controls were enrolled in this study. Most of the BD patients received mood stabilizer treatment, including valproic acid (*n* = 14, 22.6%), valproic acid plus antipsychotics (*n* = 23, 35.9%), lithium (*n* = 2, 0.03%), and lithium plus antipsychotics (*n* = 8, 12.5%). Only one patient received valproic acid plus lithium, and one received valproic acid plus lithium plus antipsychotics; eight patients (12.5%) received only antipsychotics, 3 (4.7%) only antidepressants, and 4 (6.3%) did not take any medication. There were no group differences in the usage of valproic acid (*p* = 0.182), olanzapine (*p* = 0.100), quetiapine (*p* = 0.832), amisulpride (*p* = 0.466), aripiprazole (*p* = 0.404), or lurasidone (*p* = 0.824); however, increased usage of lithium (*p* = 0.013), risperidone (*p* = 0.008) and clozapine (*p* = 0.018) was observed in the BD I patients (Table 1).

**Table 1:** Demographic data and baseline information.

	BD I (*n* = 28)	BD II (*n* = 36)	Controls (*n* = 66)	*p* value
Age, years	38.04 ± 13.49 [34.00, 22.00]	35.47 ± 12.05 [35.00, 20.75]	32.36 ± 9.85 [30.50, 17.00]	0.154
Gender, female (%)	16 (57%)	22 (61%)	38 (58%)	0.930
Smoking	2 (7.1%)	8 (22.2%)	6 (9.1%)	0.100
Body mass index^a^	25.23 ± 4.35 [24.27, 5.45]	26.21 ± 5.77 [25.64, 10.10]	24.93 ± 5.41 [23.43, 8.56]	0.516
Education, years^b^	14.22 ± 2.13 [14.00, 4.00]	14.81 ± 2.14 [16.00, 2.75]	16.26 ± 2.19 [16.00, 2.25]	0.001
RLCQ score^c^	12.39 ± 15.41 [5.00, 17.00]	13.23 ± 10.26 [13.00, 17.00]	10.28 ± 7.58 [8.00, 10.00]	0.436
HDRS score^d^	3.71 ± 5.44 [2.00, 7.25]	5.00 ± 4.93 [3.50, 7.00]	1.59 ± 1.91 [1.00, 3.00]	0.003
YMRS score^e^	2.00 ± 3.80 [0.00, 1.75]	1.18 ± 1.78 [0.00, 2.00]	0.02 ± 0.13 [0.00, 0.00]	< 0.001
Leptin (ng/mL)^f^	20.86 ± 21.03 [12.58, 13.04]	20.41 ± 17.84 [16.72, 11.55]	18.00 ± 13.64 [13.97, 13.25]	0.791
hs-CRP (mg/L)^g^	0.41 ± 0.59 [0.25, 0.36]	0.37 ± 0.56 [0.11, 0.44]	0.29 ± 0.39 [0.15, 0.33]	0.605
**Medication used**				
Valproic acid	14 (54%)	24 (71%)		0.182
Olanzapine	2 (8%)	0 (0%)		0.100
Quetiapine	7 (27%)	10 (29%)		0.832
Amisulpride	1 (4%)	3 (9%)		0.466
Aripiprazole	3(12%)	2(6%)		0.404
Lurasidone	1 (4%)	1 (3%)		0.824
Lithium	9 (35%)	3 (9%)		0.013
Risperidone	8 (32%)	2 (6%)		0.008
Clozapine	4 (15%)	0 (0%)		0.018

The data are presented as the means ± SD [median, interquartile range].

^a^Four BD I patients and 1 BD II patient without body mass index data were excluded from this calculation.

^b^Five BD I patients and 4 controls without education level data were excluded from this calculation.

^c^Five BD I patients, 1 BD II patient and 5 controls did not complete the Recent Life Change Questionnaire (RLCQ) and were excluded from this calculation.

^d^Four BD I patients, 4 BD II patients and 5 controls did not complete the 17-item Hamilton Depression Rating Scale (HDRS) and were excluded from this calculation.

^e^Four BD I patients, 2 BD II patients and 4 controls did not complete the 11-item Young Mania Rating Scale (YMRS) and were excluded from this calculation.

^f^Five BD I patients, 2 BD II patients and 12 controls did not undergo plasma leptin level measurement and were excluded from this calculation.

^g^Four BD I patients, 2 BD II patients and 3 controls did not undergo plasma CRP level measurement and were excluded from this calculation.

There were no significant differences in the demographic data of all groups, with the exception of education level (Table 1); both the BD I and BD II patients had lower education levels according to the post-hoc two-sample *t*-test. The BD I patients in this study scored 3.71 ± 5.44 on the 17-item Hamilton Depression Rating Scale (HDRS) and 2.00 ± 3.80 on the 11-item Young Mania Rating Scale (YMRS) (Table 1), and 17 (60.7%) were euthymic (scores of fewer than 7 on the HDRS and YMRS). The BD II patients scored 5.00 ± 4.93 on the HDRS and 1.18 ± 1.78 on the YMRS (Table 1), and 23 (63.9%) were euthymic. Nevertheless, both the BD I and BD II patients, in comparison with the healthy controls, demonstrated higher HDRS and YMRS scores according to the post-hoc two-sample *t*-test.

### Correlation analyses

There were no significant differences in BMI, plasma leptin level or CRP level between groups (Table 1). The plasma leptin level was sub-significantly correlated with the CRP level among the healthy controls (*r* = 0.264, *p* = 0.054) and BD II patients (*r* = 0.527, *p* = 0.002), but this was not the case in the BD I patients (*r* =  − 0.006, *p* = 0.978). Similarly, BMI was significantly correlated with the CRP level among the healthy controls (*r* = 0.466, *p* = 0.000) and BD II patients (*r* = 0.708, *p* = 0.000), but not in the BD I patients (*r* = 0.085, *p* = 0.700). In contrast, both the BD I and BD II patients exhibited significant correlations between the plasma leptin level and BMI (*r* = 0.432/0.472, *p* = 0.040/0.006, respectively); however, the healthy controls showed a sub-significant correlation (*r* = 0.263, *p* = 0.055).

### Leptin and corticostriatal circuitry connectivity

**Figure 1:**
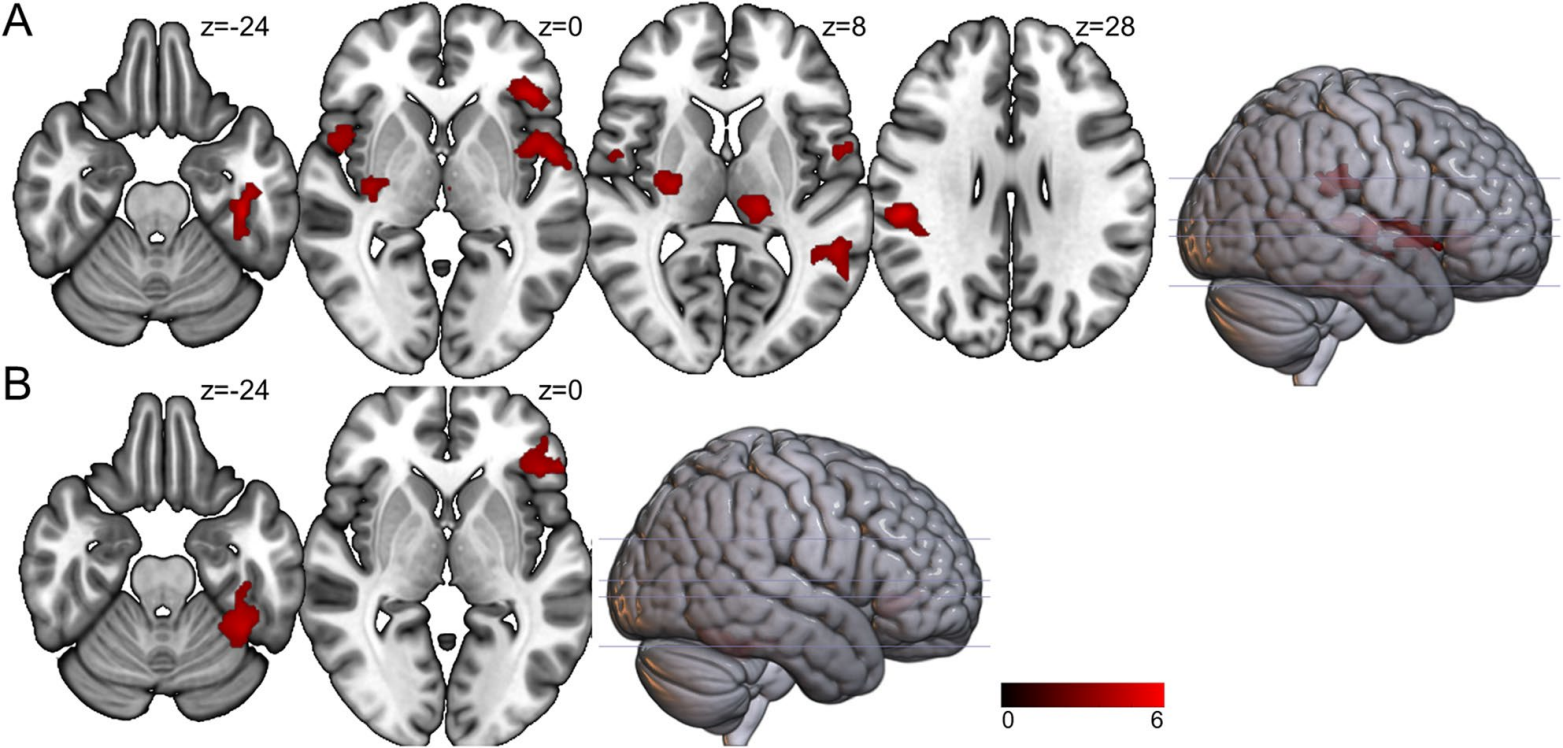
Brain regions whose functional connectivity with the left dorsal caudate was correlated with the plasma leptin level, with between-group differences. The left dorsal caudate-seeded functional connectivity was significantly correlated with the plasma leptin level among the patients with (**A**) bipolar I disorder and (**B**) bipolar II disorder. The coordinates of the peak voxel are presented in Table 2. Significance was thresholded at the uncorrected voxel level *p* = 0.001, followed by the FWE-corrected cluster level *p* = 0.05. The color bar denotes the *t*-scores. Figures are displayed according to neurological convention (left = left).

Among the healthy controls, the dorsal caudate (DC)-ventrolateral prefrontal cortex (vlPFC), DC-inferior temporal gyrus and DC-putamen FC were negatively correlated with the plasma leptin level (*r* =  − 0.597, *p* < 0.001; *r* =  − 0.532, *p* < 0.001; *r* =  − 0.345, *p* = 0.011) (Table 2, Fig. 1). In contrast, the DC-putamen FC was positively correlated with the plasma leptin level in the BD I patients (*r* = 0.609, *p* = 0.002), while a significant positive correlation between plasma leptin level and the DC-inferior temporal gyrus FC was observed in the BD II patients (*r* = 0.362, *p* = 0.035).

**Table 2:** Functional connectivity of the left dorsal caudate co-varying with leptin, with between-group differences.

Contrast	Region	Cluster	BA	*t* score	Peak coordinates		
					*x*	*y*	*z*
BD I > controls	Posterior parietal cortex	223	40	5.53	52	− 28	28
	Premotor cortex	615	6	5.29	− 54	0	2
	Ventrolateral prefrontal cortex	–	45	5.05	− 44	30	0
	Premotor cortex	243	6	4.03	52	0	6
	Inferior temporal gyrus	189	20	5.22	− 42	− 28	− 24
	Middle temporal gyrus	148	21	4.42	− 54	− 46	8
	Thalamus	160	–	4.67	− 16	− 24	6
	Putamen	192	–	4.50	26	− 14	8
BD II > controls	Ventrolateral prefrontal cortex	250	47	5.35	− 44	32	− 2
	Inferior temporal gyrus	418	20	5.12	− 46	− 42	− 28

Peak coordinates refer to the Montreal Neurological Institute (MNI) space.

No higher correlation was found in the healthy controls.

Five BD I patients, 2 BD II patients and 12 controls did not undergo plasma leptin level measurement and were excluded from this analysis.

*BA* Brodmann area, *BD* bipolar disorder.

Significance was thresholded at the uncorrected voxel level *p* = 0.001, followed by the FWE-corrected cluster level *p* = 0.05.

The right DC-seeded FC analyses yielded similar results (Supplemental Table S1). The ventral rostral putamen and the dorsal caudal putamen-seeded FC analyses yielded similar results only in the BD II patients (Supplemental Table S2), and not in the BD I patients. These supplementary analyses supported the specificity of the DC circuitry among the BD patients. Furthermore, after regressing out the effects of BMI, age and CRP, the correlations between leptin and corticostriatal connectivity yielded similar results (Supplemental Tables S3, S4).

### Between-group differences in corticostriatal circuitry connectivity

After identifying associations between plasma leptin, CRP and corticostriatal connectivity (Table 2, Fig. 1), we compared the between-group differences in corticostriatal circuitry connectivity (Table 3) to further investigate the potential effects of leptin in the BD patients.

**Table 3:** Peak MNI coordinates for the regions exhibiting significant resting-state functional connectivity with the left dorsal caudate, with between-group differences.

Contrast	Region	Cluster	BA	*t* score	Peak coordinates		
					*x*	*y*	*z*
BD I < controls	Putamen	254	–	4.91	− 32	− 16	− 6
	Putamen	175	–	4.80	− 28	− 20	10
	Parahippocampal gyrus	317	–	3.74	24	− 34	− 6
	Putamen	–	–	3.70	30	− 12	− 4
BD II < controls	Inferior temporal gyrus	1550	20	5.24	40	− 16	− 28
	Middle temporal gyrus	–	21	5.18	66	− 22	− 8
	Inferior temporal gyrus	2009	20	5.08	− 54	− 32	− 20
	Parahippocampal gyrus	–	–	4.92	− 28	− 16	− 32
	Dorsolateral prefrontal cortex	787	9	5.23	− 36	22	34
	Dorsolateral prefrontal cortex	357	9	4.04	28	30	28
	Posterior parietal cortex	1715	7	4.83	− 8	− 70	54
	Posterior parietal cortex	235	7	3.96	38	− 50	50
	Supplementary motor area	245	6	4.19	14	20	52
	Pons	509	–	5.18	6	− 26	− 28
	Orbitofrontal cortex	979	11	5.15	12	54	− 20
	Ventrolateral prefrontal cortex	–	47	4.91	50	24	− 12
	Orbitofrontal cortex	1554	11	4.87	− 26	62	− 14
	Thalamus	155	–	4.36	8	− 16	8
	Thalamus	199	–	3.41	− 14	− 18	10
	Cerebellum_Crus1	283	–	4.36	24	− 68	− 34
BD I > BD II	Orbitofrontal cortex	212	11	4.72	10	58	− 16

Peak coordinates refer to the Montreal Neurological Institute (MNI) space.

*BA* Brodmann area, *BD* bipolar disorder.

Significance was thresholded at the uncorrected voxel level *p* = 0.001, followed by the FWE-corrected cluster level *p* = 0.05.

The BD I patients exhibited significantly decreased FC between the DC and the putamen and parahippocampal gyrus as compared with the healthy controls, and between the DC and the orbitofrontal cortex (OFC) as compared with the BD II patients (Table 3). The BD II patients showed decreased FC between the DC and the OFC, vlPFC, dorsolateral prefrontal cortex, posterior parietal cortex, inferior temporal gyrus, parahippocampal gyrus, supplementary motor area, thalamus, pons, and cerebellum as compared with the healthy controls (Table 3).

## Discussion

In line with a previous meta-analysis study, our results showed that the leptin level was not altered in the BD patients as compared with the healthy controls^2^. Interestingly, the relationship between leptin and CRP found in the controls and BD II patients was absent in the BD I patients, indicating that higher CRP levels in BD I patients might induce leptin resistance^33^. Moreover, such dysregulation may arise from obesogenic diets, medications and/or BD per se^34,35^. Although we still cannot conclude whether the pathophysiological processes induced by leptin or CRP are enhanced or redundant, their possible additive or even synergistic effects need to be taken into consideration, as both leptin and CRP might increase simultaneously in BD. Our results also implied vulnerability to inflammatory and metabolic diseases in BD I patients.

Network-based studies have identified core network functional abnormalities in different states of BD^10^. The results here were in line with a unified model, suggesting that emotional dysregulation in BD arises from dynamic alterations in circuits that involve the perception of interoception^36^. In the current study, we demonstrated that there were significant correlations between the level of leptin and the corticostriatal circuitry in healthy controls, indicating a leptin-modulated effect on the interoceptive reward circuitry^37,38^. The results showed that the negative correlation between leptin and DC-seeded FC was reversed in both the BD I and BD II patients, implying that the aforementioned leptin-modulated corticostriatal circuitry was dysregulated. Furthermore, the dysregulated regions involved the emotion-regulation circuitry, including the vlPFC (Table 2, Fig. 1), as has been well-documented in BD patients^11,36^. The interaction between affective and interoceptive reward circuitries might explain the high vulnerability to metabolic syndrome of patients with BD^2,36,39^. Evidence has also shown that changes in BMI and insulin sensitivity are associated with brain intrinsic functional reorganization^40^. In BD patients, whether central leptin resistance plays a key role in leading to dysregulated corticostriatal circuits and affects food choice requires further investigation^41–43^.

The results showed that the BD I patients exhibited altered DC-putamen FC. The putamen is involved in reward-seeking and motivation behaviors, including food-seeking, and its FC is associated with childhood BMI^44^. The local striatal circuitry plays a critical role in the computing of sensory, motor and limbic information into behavioral and cognitive outputs, and impaired function in terms of an amplifier or filter may induce dysfunction of lateral inhibition^45^. Such lateral inhibition is modulated by dopamine, and inhibitory imbalance is a predominant theory in psychosis, which is only manifested in BD I, and not BD II^45,46^.

In contrast, the BD II patients showed diffuse corticostriatal hypo-connectivity, including the dorsolateral prefrontal cortex and parahippocampal gyrus, which is important for reward and decision-making in terms of food choices^47^. Furthermore, the BD II patients exhibited DC-OFC hypo-connectivity that was significantly lower than that in the BD I patients (Table 3), and showed significant dysregulation of leptin in the DC-vlPFC FC (Table 2, Fig. 1). Obese humans exhibit vlPFC-OFC hyper-connectivity, which has a substantial trend in terms of a negative correlation with the level of leptin^48^, while leptin injections may down-regulate sensitivity to food via hypothalamus-OFC hypo-connectivity^49^. As the OFC is involved in emotional processes, while the vlPFC encodes certainty and predictions^36^, our results further supported that BD II is not a milder form of BD I, but presents with wide-ranging functional abnormalities in the reward circuitry^11,50,51^.

Our study had limitations in terms of the cross-sectional design and in not considering eating habits. This should be taken into account in subsequent study. Given that the BD patients may have been a number of variables that could confound the observed associations; nevertheless, after regressing out the effects of BMI, age and CRP, our main findings remained robust (Supplemental Tables S3, S4).

## Conclusions

The results of this study connected circuits controlling mood and energy balance with corticostriatal circuitry^52^. Moreover, the dysregulated circuitry in BD implied vulnerability to inflammatory and metabolic diseases, especially in BD I. These results provided insights that could kindle hope for the development of novel circuitry-based treatments for BD^53^.

## Methods

### Ethics approval and consent to participate

All participants provided their written informed consent. The study was approved by the Institutional Review Board of National Cheng Kung University Hospital and was conducted in accordance with the Declaration of Helsinki.

### Subjects

All patients were recruited from the psychiatric outpatient department at National Cheng Kung University Hospital, while the healthy controls were recruited from the community through advertisement. All participants, who were either Mandarin or Taiwanese speakers, were screened by a psychiatrist using the Chinese version of the Mini International Neuropsychiatry Interview (MINI)^54^, the HDRS^55,56^, and the YMRS. All patients were diagnosed by a psychiatrist to determine eligibility according to the Diagnostic and Statistical Manual of Mental Disorders, Fifth Edition (DSM-5). A proportion of the subjects (BD I: *n* = 15, 54%; BD II: *n* = 25, 69%; healthy controls: *n* = 48, 73%) overlapped with those enrolled in our previous published studies^28,57,58^.

The exclusion criteria for all the participants were: (1) any psychiatric (other than BD and tobacco use disorder), neurological, autoimmune, serious surgical, or severe physical illnesses, such as acute coronary syndrome, kidney dialysis, hepatic failure, or transplant; (2) any head injury history with loss of consciousness; (3) any medications that could affect the immune system or use of anti-inflammatory medications; (4) any contraindications in relation to magnetic resonance imaging (MRI), such as having a metal implant, a pacemaker implant, or claustrophobia; (5) plans for pregnancy or a positive pregnancy test.

### Experimental design

After enrolment in the study, all patients received treatment as usual, and the administration of medications was recorded. There was no significant relapse of BD that necessitated clinical care changes during the study. All participants in the three groups completed the Recent Life Change Questionnaire (RLCQ) to assess the type and magnitude of life events during the previous 12 months^59^. The RLCQ, which consists of 39 items querying representative life change events, is the Taiwanese version of the Schedule of Recent Experience^60^, which consisted originally of 43 life change events, later termed life change units^61^. Blood samples were taken for the measurement of fasting plasma leptin and CRP levels, and FC was examined during the resting state using functional magnetic resonance imaging (fMRI). For detailed information on image acquisition, image preprocessing, definition of the seeds in the left DC and other areas among the basal ganglia (for additional testing to examine specificity), and seed-based FC maps, please refer to our published papers^57,58^.

### Levels of fasting plasma leptin and high-sensitivity C-reactive protein

The participants were instructed to fast for at least 9 h prior to each examination. Blood samples for the leptin and CRP assays were collected between 08:00 and 10:00 am in 5-mL EDTA tubes and stored at 4 °C in a fridge. Plasma was isolated by centrifugation at 1800×*g* for 15 min at 4 °C and immediately stored at − 80 °C. The levels of fasting plasma leptin (Linco Research, St Louis, MO, USA) and CRP (eBioscience, San Diego, CA, USA) were measured using ELISA methods. For detailed information on the catalog numbers, minimum detectable levels, and intra- and inter-assay coefficients of the ELISA kits used for the measurement of leptin and CRP, please see Supplemental Table S5. Dilute hs-CRP samples from 1:30 to 1:1000 with Assay Buffer.

### Statistical analyses

SPSS Statistics 20.0 (SPSS Inc., Chicago, IL, USA) was used for all analyses. Missing data were excluded from the calculations, and the numbers of participants with missing data are indicated in the footnote of each table. Results were considered significant at *p* < 0.05 (two-tailed). As most of the variables shown in Table 1 were not normally distributed in a Gaussian manner, a Kruskal–Wallis H test was conducted to examine between-group (BD I patients, BD II patients and healthy controls) differences in the demographic characteristics, HDRS score, YMRS score, life events, and plasma levels of leptin and CRP. The *post-hoc* Mann–Whitney U test was performed whenever the between-group difference was significant. Pearson correlation analyses were performed to test the relationships among the plasma leptin level, CRP level, and BMI.

### Image analysis

One-way ANOVA was employed to analyze the FC maps using SPM12 (Wellcome Trust Centre for Neuroimaging, London, https://www.fil.ion.ucl.ac.uk/spm/). Statistical maps were computed to identify changes in the DC-seeded FC for between-group comparisons. Significance was thresholded at the uncorrected voxel-level *p* = 0.001, followed by the voxel-level family-wise error (FWE) rate-corrected cluster-level at *p* = 0.05 for whole-brain multiple comparisons.

Another one-way ANOVA was performed to determine the correlations between the DC-seeded FC and the leptin level. We entered the demeaned (in SPM12) values as regressors to identify brain regions exhibiting higher correlations with the DC-seeded FC among the three groups. Significance was thresholded at the uncorrected voxel-level *p* = 0.001, followed by the FWE-corrected cluster-level at *p* = 0.05.

As SPM12 reports peak coordinates as identified within a confluent cluster, there can be multiple peaks that sit on different brain regions/areas (e.g., Brodmann area). We reported one representative peak for each region/area. To display 3D imaging, we used MRIcroGL for 3D rendering (Department of Psychology, University of South Carolina http://www.mccauslandcenter.sc.edu/mricrogl). To show the regression results in scatterplots, we extracted the DC-seeded FC values in each brain region showing higher correlations with the DC-seeded FC among the three groups (peak MNI coordinates are presented in Tables 2, 3, radius = 3 mm). The corresponding correlation coefficients (*r*) and *p* values were analyzed using SPSS Statistics 20.0.

## Supplementary Information

Supplementary Tables.

## Data Availability

The datasets used and/or analyzed during the current study are available from the corresponding author on reasonable request.
