# Supplementary material for: Associations of leptin and corticostriatal connectivity in bipolar disorder
Source: Sci Rep. 2022 Dec 19;12:21898. doi: 10.1038/s41598-022-26233-8 (PMC9763246; doi:10.1038/s41598-022-26233-8)
Supplement: Supplementary file 1 — Supplementary Tables. [file 41598_2022_26233_MOESM1_ESM.docx]

**Associations of leptin and corticostriatal connectivity in bipolar disorder**

Shyh-Yuh Wei, Huai-Hsuan Tseng, Hui Hua Chang, [Wei Hung Chang](https://researchoutput.ncku.edu.tw/zh/persons/wei-hung-chang),

Yen Kuang Yang, Po See Chen

**Supplemental Table S1.** Functional connectivity of the right dorsal caudate co-varying with leptin, with between-group differences.

|  |  |  |  |  | Peak coordinates | | | |
| --- | --- | --- | --- | --- | --- | --- | --- | --- |
| Contrast | Region | Cluster | BA | *t* Score | *x* | *y* | *z* |  |
| BD I > Controls | Posterior parietal cortex | 204 | 40 | 4.64 | 50 | -26 | 26 |  |
|  | Ventrolateral prefrontal cortex | 173 | 45 | 5.58 | -42 | 30 | 0 |  |
|  | Inferior temporal gyrus | 171 | 20 | 4.95 | -40 | -26 | -24 |  |
|  | Thalamus | 165 | — | 4.16 | -22 | -24 | 4 |  |
|  | Insula | 603 | 13 | 4.49 | 46 | 8 | -4 |  |
|  | Premotor cortex | — | 6 | 4.46 | 48 | 0 | 4 |  |
|  | Putamen | — | — | 3.73 | 32 | -10 | 6 |  |
| BD II > Controls | Ventrolateral prefrontal cortex | 181 | 47 | 5.79 | -46 | 28 | -2 |  |
|  | Inferior temporal gyrus | 409 | 20 | 6.10 | -46 | -42 | -28 |  |
|  | Premotor cortex | 261 | 6 | 3.75 | 60 | 4 | 4 |  |
|  | Premotor cortex | 199 | 6 | 4.20 | 30 | -2 | 62 |  |
|  | Premotor cortex | 192 | 6 | 4.09 | -54 | 2 | 0 |  |
|  | Supplementary motor area | 218 | 6 | 4.54 | 2 | -8 | 72 |  |
|  | Posterior parietal cortex | 147 | 40 | 3.72 | -64 | -22 | 18 |  |

Peak coordinates refer to the Montreal Neurological Institute (MNI) space. Significance was thresholded at the uncorrected voxel level *p* = 0.001, followed by the FWE-corrected cluster level *p* = 0.05.

No higher correlation was found in the healthy controls.

BA: Brodmann area; BD: bipolar disorder.

Five BD I patients, 2 BD II patients and 12 controls did not undergo plasma leptin level measurement and were excluded from this analysis.

**Supplemental Table S2.** Functional connectivity of the lentiform nucleus, ventral rostral putamen and dorsal caudal putamen co-varying with leptin, with between-group differences.

|  |  |  |  |  |  | Peak coordinates | | | |
| --- | --- | --- | --- | --- | --- | --- | --- | --- | --- |
| Seed | Contrast | Region | Cluster | BA | *t* Score | *x* | *y* | *z* |  |
| L lentiform nucleus | BD I > Controls | Orbitofrontal cortex | 164 | 11 | 4.22 | -22 | -16 | -26 |  |
|  |  | Putamen | — | — | 5.58 | -18 | 8 | -10 |  |
| R lentiform nucleus | BD II > Controls | Brainstem | 156 | — | 3.58 | -6 | -20 | -18 |  |
| L ventral rostral putamen | BD II > Controls | Ventrolateral prefrontal cortex | 439 | 47 | 5.57 | -42 | 30 | -4 |  |
|  |  | Ventrolateral prefrontal cortex | 471 | 44 | 4.49 | -42 | 10 | 12 |  |
|  |  | Ventrolateral prefrontal cortex | 178 | 47 | 3.92 | 40 | 42 | -2 |  |
|  |  | Inferior temporal gyrus | 199 | 37 | 4.79 | 68 | -48 | -12 |  |
|  |  | Inferior temporal gyrus | 192 | 37 | 4.12 | -56 | -62 | -20 |  |
|  |  | Frontal eye fields | 323 | 8 | 5.43 | -4 | 36 | 42 |  |
|  |  | Middle occipital gyrus | 154 | 19 | 4.29 | -28 | -88 | 30 |  |
| R ventral rostral putamen | BD II > Controls | Ventrolateral prefrontal cortex | 344 | 47 | 4.91 | -42 | 30 | -4 |  |
|  |  | Ventrolateral prefrontal cortex | 290 | 44 | 4.00 | -34 | 16 | 26 |  |
|  |  | Ventrolateral prefrontal cortex | 176 | 47 | 4.16 | 44 | 44 | -4 |  |
|  |  | Fusiform gyrus | 197 | 37 | 4.44 | -34 | -40 | -24 |  |
|  |  | Posterior parietal cortex | 173 | 39 | 4.21 | -38 | 60 | 40 |  |
| L dorsal caudal putamen | BD II > Controls | Ventrolateral prefrontal cortex | 685 | 47 | 4.79 | 40 | 42 | -2 |  |
|  |  | Ventrolateral prefrontal cortex | 491 | 45 | 4.64 | -42 | 18 | 10 |  |
|  |  | Ventrolateral prefrontal cortex | 723 | 46 | 4.56 | -42 | 40 | 4 |  |
|  |  | Fusiform gyrus | 183 | 37 | 4.28 | 66 | -44 | -12 |  |
|  |  | Posterior parietal cortex | 385 | 39 | 4.71 | 48 | -60 | 26 |  |
|  |  | Frontal eye fields | 1001 | 8 | 5.25 | -4 | 38 | 40 |  |
|  |  | Frontal eye fields | 257 | 8 | 4.47 | -24 | 26 | 42 |  |
|  |  | Frontal eye fields | 386 | 8 | 4.09 | 38 | 20 | 42 |  |
|  |  | Middle occipital gyrus | 382 | 19 | 4.16 | -30 | -88 | 30 |  |
| R dorsal caudal putamen | BD II > Controls | Ventrolateral prefrontal cortex | 773 | 47 | 4.74 | -44 | 30 | -4 |  |
|  |  | Orbitofrontal cortex | 983 | 47 | 5.30 | 34 | 44 | -12 |  |
|  |  | Orbitofrontal cortex | 178 | 11 | 4.17 | -20 | 24 | -16 |  |
|  |  | Posterior parietal cortex | 544 | 39 | 4.49 | -40 | -62 | 40 |  |
|  |  | Posterior parietal cortex | 151 | 39 | 3.95 | 46 | -58 | 24 |  |
|  |  | Frontal eye fields | 338 | 8 | 4.15 | 38 | 18 | 48 |  |
|  |  | Frontal eye fields | 489 | 8 | 4.05 | 0 | 38 | 48 |  |
|  |  | Hippocampus | 183 | — | 4.70 | -24 | -40 | 2 |  |
|  |  | Posterior cingulate cortex | 210 | 31 | 4.77 | 10 | -58 | 28 |  |
|  |  | Posterior cingulate cortex | 151 | 23 | 4.16 | -6 | -44 | 24 |  |

Peak coordinates refer to the Montreal Neurological Institute (MNI) space. Significance was thresholded at the uncorrected voxel level *p* = 0.001, followed by the FWE-corrected cluster level *p* = 0.05.

No higher correlation was found in the healthy controls.

BA: Brodmann area; BD: bipolar disorder.

Five BD I patients, 2 BD II patients and 12 controls did not undergo plasma leptin level measurement and were excluded from this analysis.

**Supplemental Table S3.** Functional connectivity of the left dorsal caudate co-varying with leptin, with between-group differences, after regressing out the effects of BMI, age and CRP.

|  |  |  |  |  | Peak coordinates | | | |
| --- | --- | --- | --- | --- | --- | --- | --- | --- |
| Contrast | Region | Cluster | BA | *t* Score | *x* | *y* | *z* |  |
| BD I > Controls | Ventrolateral prefrontal cortex | 166 | 45 | 5.13 | -44 | 30 | 0 |  |
|  | Inferior temporal gyrus | 157 | 20 | 5.07 | -42 | -28 | -24 |  |
|  | Premotor cortex | 403 | 6 | 5.16 | -54 | 0 | 2 |  |
|  | Insula | — | 13 | 4.99 | -44 | 6 | 2 |  |
|  | Insula | 199 | 13 | 4.69 | 46 | 8 | -2 |  |
|  | Thalamus | 151 | — | 4.58 | -16 | -24 | 6 |  |
|  | Posterior parietal cortex | 196 | 40 | 3.90 | 44 | -34 | 22 |  |
| BD II > Controls | Ventrolateral prefrontal cortex | 321 | 47 | 5.23 | -42 | 32 | -4 |  |
|  | Ventrolateral prefrontal cortex | 156 | 44 | 4.38 | 54 | 10 | 0 |  |
|  | Inferior temporal gyrus | 611 | 20 | 5.21 | -46 | -42 | -28 |  |
|  | Substantia nigra | 259 | — | 5.17 | 8 | -22 | -16 |  |

Peak coordinates refer to the Montreal Neurological Institute (MNI) space. Significance was thresholded at the uncorrected voxel level *p* = 0.001, followed by the FWE-corrected cluster level *p* = 0.05.

No higher correlation was found in the healthy controls.

BA: Brodmann area; BD: bipolar disorder.

Five BD I patients, 2 BD II patients and 12 controls did not undergo plasma leptin level measurement and were excluded from this analysis.

**Supplemental Table S4.** Functional connectivity of the right dorsal caudate co-varying with leptin, with between-group differences, after regressing out the effects of BMI, age and CRP.

|  |  |  |  |  | Peak coordinates | | | |
| --- | --- | --- | --- | --- | --- | --- | --- | --- |
| Contrast | Region | Cluster | BA | *t* Score | *x* | *y* | *z* |  |
| BD I > Controls | Posterior parietal cortex | 184 | 40 | 4.57 | 50 | -26 | 26 |  |
|  | Ventrolateral prefrontal cortex | 176 | 45 | 5.46 | -42 | 30 | 0 |  |
|  | Insula | 243 | 13 | 4.41 | 46 | 8 | -4 |  |
|  | Insula | 295 | 13 | 3.98 | 36 | -16 | 8 |  |
|  | Putamen | — | — | 3.56 | 32 | -2 | -2 |  |
| BD II > Controls | Ventrolateral prefrontal cortex | 148 | 47 | 5.45 | -46 | 28 | -2 |  |
|  | Inferior temporal gyrus | 564 | 20 | 6.07 | -46 | -42 | -28 |  |
|  | Premotor cortex | 210 | 6 | 4.43 | 28 | -4 | 64 |  |
|  | Supplementary motor area | 262 | 6 | 4.76 | 2 | -6 | 72 |  |
|  | Superior temporal gyrus | 152 | 22 | 4.39 | -54 | 10 | -8 |  |
| BD II > BD I | Cerebellum | 269 | — | 5.23 | 12 | -74 | -40 |  |

Peak coordinates refer to the Montreal Neurological Institute (MNI) space. Significance was thresholded at the uncorrected voxel level *p* = 0.001, followed by the FWE-corrected cluster level *p* = 0.05.

No higher correlation was found in the healthy controls.

BA: Brodmann area; BD: bipolar disorder.

Five BD I patients, 2 BD II patients and 12 controls did not undergo plasma leptin level measurement and were excluded from this analysis.

**Supplemental Table S5.** ELISA kits used for the measurement of leptin and CRP.

| **ELISA kit** | **Human Leptin 96-Well Plate Assay** | **CRP Human Instant ELISA** |
| --- | --- | --- |
| Catalog number | EZHL-80SK | Cat #BMS288INST |
| Assay range | 0.78–100 ng/mL | 78–5,000 pg/mL |
| Inter-assay CV | 2.6–6.2%, Sensitivity Assay 1.3–8.6 | 13.10% |
| Intra-assay CV | 2.6–4.6%, Sensitivity Assay 1.4–4.9 | 6.90% |
